# Development and validation of a novel blood-based biomarker for gastric cancer triage in chronic dyspepsia

**DOI:** 10.1038/s41746-026-02618-1

**Published:** 2026-04-17

**Authors:** Minji Seo, Ka Man Cheung, Serene J. L. Lam, Peter Y. M. Woo, Winnie W. Y. Sung, James C. H. Chow, Ada S. M. Yip, Stephen K. K. Ng, Martin S. C. Lee, Henry H. W. Liu, Daisy M. Y. Kan, Sau Shan Kao, Harry H. Y. Yiu, David C. C. Lam

**Affiliations:** 1 Department of Mechanical and Aerospace Engineering, The Hong Kong University of Science and Technology, Hong Kong, China; 2 Department of Clinical Oncology, Queen Elizabeth Hospital, Hong Kong, China; 3 Department of Internal Medicine, United Christian Hospital, Hong Kong, China; 4 Department of Neurosurgery, Prince of Wales Hospital, Hong Kong, China; 5 Department of Surgery, Kwong Wah Hospital, Hong Kong, China; 6 Department of Surgery, Prince of Wales Hospital, Hong Kong, China; 7 Department of Surgery, Queen Elizabeth Hospital, Hong Kong, China; 8 Department of Medicine, Queen Elizabeth Hospital, Hong Kong, China

**Keywords:** Biomarkers, Cancer, Diseases, Gastroenterology, Medical research, Oncology

## Abstract

Global implementation of gastric cancer (GC) screening in chronic dyspepsia populations faces challenges due to the high number-needed-to-scope (NNS) for oesophagogastroduodenoscopy. Routine blood tests (RBT) have limited utility for GC screening but offer potential for risk stratification when repurposed through machine learning. This study develops and validates a machine-learning-integrated biomarker (RBT-GC) that uses opportunistic triage to optimise endoscopy resource allocation. The team analysed 20 years of territory-wide retrospective data (2000–2020) from the Hong Kong Hospital Authority. 24 RBT and demographic features from 210,463 subjects (3071 cases) between 2000 and 2015 were used in training. An independent cohort of 90,479 subjects (2066 cases) from 2016 to 2020 was used in validation. The RBT-GC model successfully stratified validation cohort (2.3% baseline GC prevalence) into low-risk (0.3% prevalence), intermediate-risk (1.9%) and high-risk (14.0%) categories. The model detected (1276 cases) 12x more than CEA (102 cases) and 30x more than CA19.9 (42 cases). The application of opportunistic RBT-GC risk stratification reduced the NNS from 44 to 7 in the high-risk category of validation cohort. This machine learning approach repurposed standard blood tests into an opportunistic, affordable, scalable triage tool to alleviate endoscopic burdens across healthcare systems.

## Introduction

Gastric cancer (GC) is a major health concern due to late-stage diagnosis and poor prognosis. It ranks 5th among the leading causes of cancer-related mortality^1^. GC incidence is high in regions like Asia, Eastern Europe and South America. The mortality-to-incidence (M/I) ratio (Supplementary Table 1), a surrogate for 5-year survival, is significantly lower (70% reduction in M/I ratio) in countries with established screening programmes. This suggests effective cancer screening has a positive impact on improving GC survival^2,3^.

Oesophagogastrodudodenoscopy (OGD) examination is highly sensitive and specific for GC detection^4–6^ and has the advantage of being both diagnostic and therapeutic for pre-malignant and predisposing conditions for GC. However, its invasive nature, substantial resource requirements^7^ and low yield in finding GC cases in general populations limit its effectiveness as the main modality of a population-based screening and the cost-effectiveness is low in countries with low incidence rate.

The challenge of low diagnostic yield necessitating a high volume of procedures is common in other screening domains. Colonoscopy for colorectal cancer screening requires up to 200 screenings to detect one case^8^. The introduction of the faecal immunochemical test in Hong Kong’s 2016 Colorectal Cancer (CRC) Screening Programme reduced the number needed to scope (NNS) from over 200–18^9^. Moreover, 57% of the detected CRC cases were found at early stage. The addition of a low-cost, non-invasive pre-screening before imaging has improved resource efficiency and sustainability of colorectal cancer screening. More recently, Nemlander et al.^10^ developed a machine learning model using diagnostic information. The CRC screening model achieved a sensitivity of 73% and specificity of 84% in this new paradigm. The success shows that adding a pre-screening test before OGD may enhance GC screening efficiency and help detect GC early.

A number of risk assessment approaches have been investigated for GC along this new paradigm. Miki et al.^11^ used Helicobacter pylori antibody and serum pepsinogen levels and developed the ABC method to predict future GC risk in general populations. The study did not target dyspepsia population. The screening results were below expectation and testing results in Chinese population showed low accuracy. Cai et al.^12^ proposed a GC risk stratification method based on PGI/II ratio, G-17 levels and lifestyle habits in a >14k high-risk Chinese population. This approach achieved 67% accuracy, but relied on specialised biomarkers. Similarly, Leung et al.^13^ also targeted high-risk GC patients who received HP eradication and employed machine learning to predict GC risk in a >89k cohort. Ishizu et al.^14^ developed a machine learning model that predicts lymph node metastasis preoperatively in GC patients with gastrectomy. So et al.^15^ used serum miRNA panels to assess GC risk non-invasively. These approaches rely on the specialised tests which limit applicability and implementation in broader populations.

An alternative strategy that leverages existing clinical data has been examined. The statistical relationships between routine blood tests (RBT) components and GC was investigated^16^. The components included complete blood count, liver/renal function test, which are alternative options for malignancy detection. The statistical analysis revealed that multivariate RBT has high diagnostic values while the diagnostic value of univariate RBT is low. Gradual declines in haemoglobin and rises in platelet count, even within clinically normal ranges, were strong indicators of stomach malignancy. A clinical big-data study including 220k individuals by our team also revealed a pattern among the RBT components which differentiate individuals with or without GC^17^. The discovery of subtle multivariate patterns, often missed by conventional statistical tests, highlights the potential of machine learning in repurposing existing clinical data into clinically meaningful biomarkers. Unlike the conventional statistical methods, tree-based models and others may capture complex, non-linear interactions between biomarkers^18,19^. This foundation enables the development of high-sensitivity methods based on RBT that were overlooked by conventional hypothesis-driven biomarkers.

**Fig. 1:**
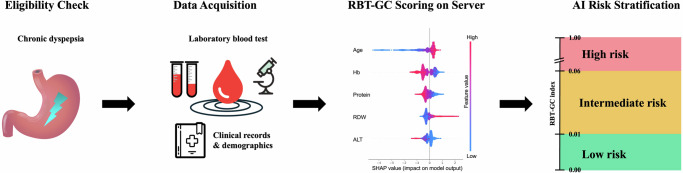
Workflow of GC risk assessment using RBT-GC biomarker. A patient with chronic dyspepsia presents to a gastrointestinal clinic, where the clinician orders routine blood test including CBC, LFT and RFT. The RBT-GC model integrates the results of 24 RBT components along with two demographic features (age and sex) to generate an individualised RBT-GC risk score. The model stratifies patients into risk groups to assist clinical decision-making, such as prioritising OGD referrals for high-risk individuals while adhering to standard clinical protocols for surveillance timing. The figure illustrates the integration of laboratory data, model-based risk assessment and corresponding clinical decision pathways. This figure was created by the authors by adapting original elements sourced from Wikimedia Commons under the Creative Commons Attribution 4.0 International.

The primary objectives of this study are to develop and validate a machine-learning-integrated, RBT-based GC biomarker for risk stratification of patients with chronic dyspepsia (Fig. 1). In this study, we evaluated the near-diagnosis triage performance of the RBT-based biomarker and compared it to that of carinoembryonic antigen (CEA) and carbohydrate antigen (CA19.9). The screening performance of the RBT-based biomarker for 6–12-month records before diagnosis is reported in a separate paper. The biomarker is developed for use in gastroenterology clinics in Hong Kong public hospitals to guide triage before endoscopy. This study examines whether RBT-based GC biomarker (RBT-GC) can support endoscopy referral decisions, thereby reducing unnecessary procedures and improving resource prioritisation.

## Results

### Study population and cohort characteristics

**Fig. 2:**
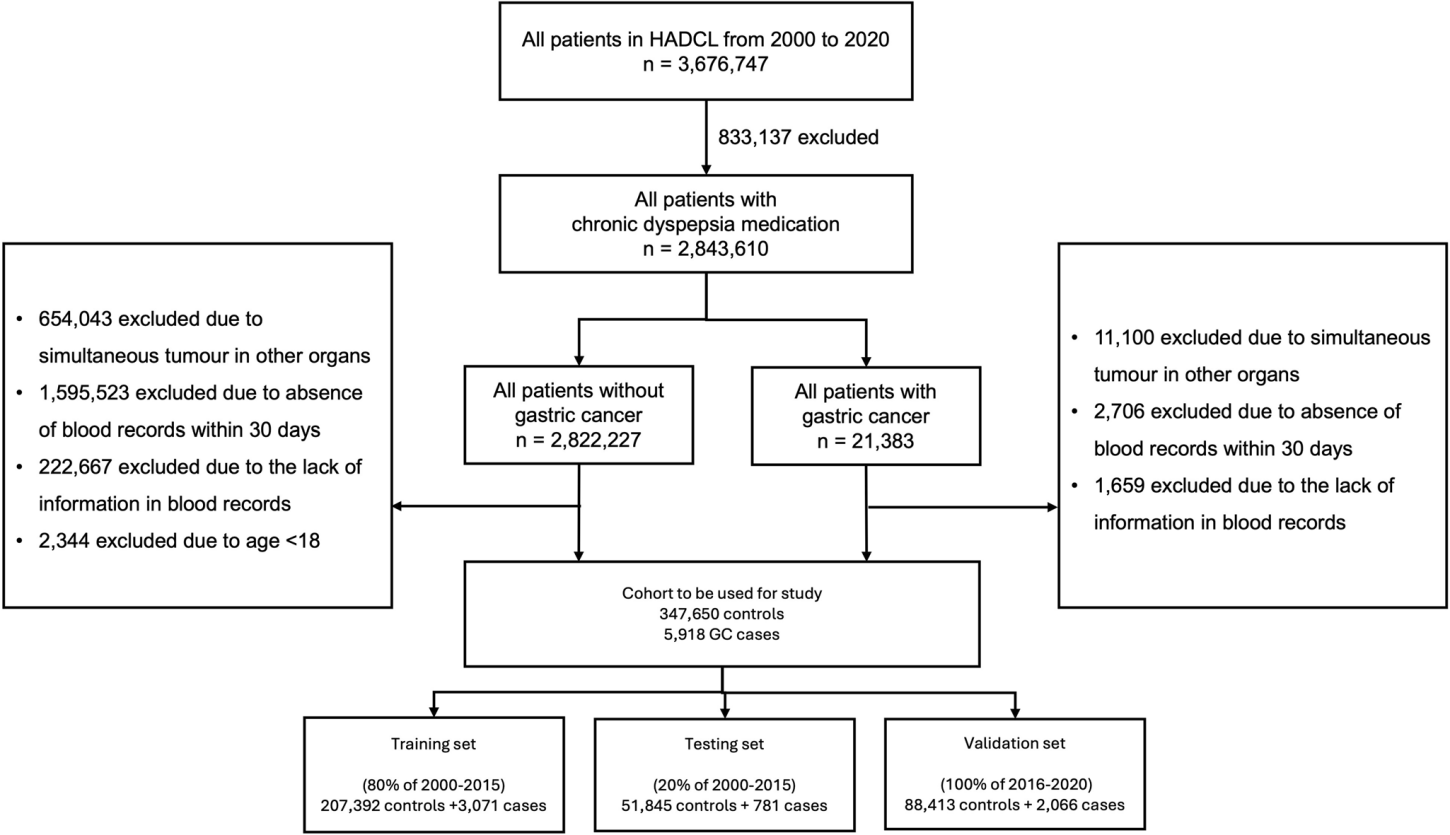
Flowchart of the RBT-GC model development and validation process. 2,843,610 patients with chronic dyspepsia were initially identified based on prescriptions for dyspepsia-related medications from 2000 to 2020. Clinically irrelevant cases were excluded, including 665,143 patients with prior or simultaneous malignancy, and 1,598,229 patients without RBT within 30 days from the pivot time. The remaining cohort was used to train, test and validate the model. The training set included 80% of patients from 2000–2015 (*n* = 210,463) for model training with six machine learning algorithms. The testing set consisted of the remaining 20% (*n* = 52,626). The model with the highest AUROC, the highest AUPRC and SHAP feature alignment with clinical findings was selected as the final RBT-GC model, which was then validated on patients from 2016 to 2020 (*n* = 90,479).

This study analysed medical data of 3,676,747 patients in the Hong Kong Hospital Authority Data Collaboration Lab (HADCL). Patients without chronic dyspepsia medications (*n* = 833,137), with simultaneous cancer at organs other than stomach (*n* = 665,143), without RBT records within the defined period (*n* = 1,598,229) and with insufficient information in RBT (*n* = 224,326) were excluded (Fig. 2). The study cohort included 5918 (1.7%) GC cases and 347,650 (98.3%) dyspeptic controls. Both groups showed similar distribution of biological sex, where 61.5% and 55.7% of cases and controls were male (Table 1). The mean age was higher in cases (73 ± 13 years) than in controls (61 ± 19 years).

**Table 1:** Patient characteristics

*N* (%)	Case *N* = 5918	Control *N* = 347,650
Age, median (IQR) in years	76 (65–83)	63 (48–77)
Sex		
Male	3638 (60)	193,629
Female	2280 (40)	154,021
Medications		
Aluminium hydroxide	2768 (47)	113,922 (33)
Simeticone	1885 (32)	50,127 (14)
Famotidine	4169 (70)	104,069 (30)
Pantoprazole	4250 (72)	163,007 (47)
Esomeprazole	2379 (40)	46,686 (13)
Rabeprazole	684 (12)	20,420 (6)
Dexlansoprazole	53 (1)	1269 (0)
Comorbidities		
Helicobacter pylori infection (B96.81)	<5 (0)	7 (0)
Anaemia (D64.9)	1442 (24)	37,872 (11)
Type 2 Diabetes Mellitus (E11)	1248 (21)	78,517 (23)
Hyperlipidaemia (E78)	868 (15)	69,222 (20)
Hypertension (I10)	2332 (39)	141,032 (41)
Chronic ischaemic heart disease (I25)	688 (12)	49,002 (14)
Atrial fibrillation (I48)	653 (11)	39,351 (11)
Congestive heart failure (I50)	672 (11)	49,837 (14)
Haemorrhagic stroke (I60-61)	120 (3)	18,196 (5)
Ischaemic stroke (I63)	402 (7)	30,917 (9)
Esophagitis (K20)	193 (3)	4891 (1)
Gastroesophageal reflux (K21)	141 (2)	4898 (1)
Gastric ulcer (K25)	1473 (25)	17,144 (5)
Duodenal ulcer (K26)	155 (3)	12,410 (4)
Gastritis (K29)	817 (14)	33,530 (10)
Cirrhosis (K74.6)	40 (1)	3729 (1)
Chronic renal failure (N18)	363 (6)	29,983 (9)

The cohort is divided into training, testing and validation sets. The training set included 210,463 patients with a case-to-control ratio of 1:68. The testing set included 52,626 patients with a ratio of 1:66. The validation set included 90,479 patients with a ratio of 1:48. Each RBT component was normalised to have lower and upper range of 0.20 and 0.80, respectively. Cases and controls from training set and validation set were statistically analysed. All features of 24 RBT components showed significant separations between cases and controls (Table 1).

### RBT-GC biomarker development

**Fig. 3:**
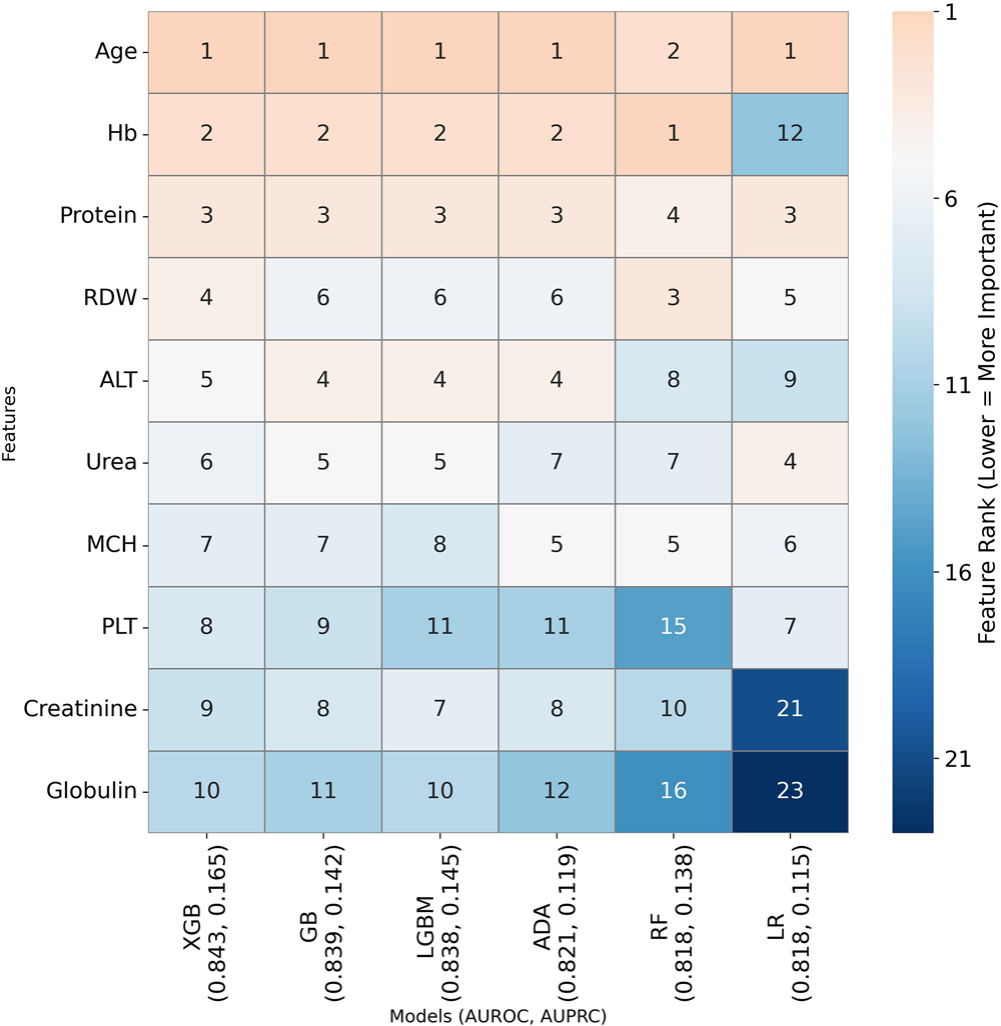
Ranked feature importance (SHAP) in models. Feature importance ranking across multiple machine learning models for predicting a GC risk. The heatmap shows feature rank (lower values indicate higher importance) across six machine learning models. Darker blue shades correspond to features with lower importance, while lighter shades indicate higher importance. Age, Hb, protein and red cell distribution width (RDW) consistently ranked as top predictors. Area under the receiver operating characteristic curve (AUROC), and area under the precision-recall curve (AUPRC) values are shown for each model. Extreme gradient boosting (XGB), gradient boost (GB), light gradient boosting machine (LGBM), adaptive boost (ADA), random forest (RF), and logistic regression (LR).

In the testing set, the Extreme gradient boosting (XGB) model outperformed other methods, achieving the highest area under the receiver operating characteristic (AUROC) of 0.843 and the higest area under the precision recall curve (AUPRC) of 0.165 (Supplementary Fig. 1). A heatmap comparing the Shapley additive explanations (SHAP) rankings of features across multiple machine learning models is shown in Fig. 3. The SHAP analysis shows that feature rankings are consistent across the models. Age, haemoglobin (Hb), protein and red blood cell distribution width (RDW) were consistently at the top of the SHAP of the models. This consistency across the models confirms the model-independent nature of the GC biomarker in RBT. Among all tested models, the XGB algorithm was selected for RBT-GC clinical validation due to its performance and the clinical interpretability of its feature rankings.

### RBT-GC biomarker validation

RBT-GC biomarker was developed using an XGB algorithm with missing values. AUROC and AUPRC in the validation cohort are 0.868 and 0.235, respectively (Supplementary Fig. 2). The calibration slope in the validation cohort was 1.01 [95% confidence interval (CI), 0.91–1.11] and intercept −0.15 [95% CI, −0.32 to 0.02], indicating good calibration (Supplementary Fig. 2). Decision Curve Analysis (DCA) confirmed that the RBT-GC model has high clinical utility across threshold probabilities from 0.00 to 0.55 (Supplementary Fig. 2).

RBT-GC biomarker demonstrated effective risk stratification within the validation cohort (Table 2). The model successfully identified 61.8% (1276/2066) of all GC cases and improved the GC prevalence from the baseline 2.3 to 13.8% in high-risk group. The model’s stability was further examined by comparing the testing cohort (>5 year follow-up) with the validation cohort (<5 year follow-up), as shown in Supplementary Table 2. The analysis revealed that AUROC has a variance of 0.03 confirming the model robustness across varying follow-up durations. A subgroup analysis of RBT-GC performance across demographics is presented in Supplementary Table 3. This robustness extends to data availability, as demonstrated by sub-models trained on sub-panels (Supplementary Table 4). The sub-models achieved AUROCs ranging from 0.75 to 0.84, comparable to the full panel’s 0.87. Subjects with incomplete data may still be evaluated without significant performance degradation.

**Table 2:** Comparison of cohort size and biomarker accuracy

	Data from 2000 to 2015	Data from 2016 to 2020		
	RBT-GC (Training)	RBT-GC (Validation)	CEA	CA19.9
Overall cohort				
Case (*N*)	3071	2066	625	222
Control (*N*)	207,392	88,413	5093	1890
NNS (Total/Case)	68.5	43.8	9.1	9.5
GC prevalence (%)	1.5	2.3	10.9	10.5
High risk (score higher than 0.06)				
Case (*N*, %)	2230 (72.6%)	1276 (61.8%)	102 (16.3%)	42 (18.9%)
Control (*N*, %)	18,807 (9.1%)	7942 (9.0%)	292 (5.7%)	167 (8.8%)
NNS (Total/Case)	9.4	7.2	3.9	5.0
GC prevalence (%)	10.6	13.8	25.9	20.1
Intermediate risk (score in between 0.01 and 0.06)				
Case (*N*, %)	788 (25.7%)	643 (31.1%)	76 (12.2%)	43 (19.3%)
Control (*N*, %)	84,033 (40.5%)	33,721 (38.1%)	661 (13.0%)	475 (25.1%)
NNS (Total/Case)	107.6	53.4	9.7	12.0
GC prevalence (%)	0.9	1.9	10.3	8.3
Low risk (score lower than 0.01)				
Case (*N*, %)	53 (1.7%)	147 (7.1%)	447 (71.5%)	137 (61.7%)
Control (*N*, %)	104,552 (40.5%)	46,750 (52.9%)	4140 (81.3%)	1248 (66.0%)
NNS (Total/Case)	1973.7	319.0	10.3	10.1
GC prevalence (%)	0.1	0.3	9.7	9.9

**Fig. 4:**
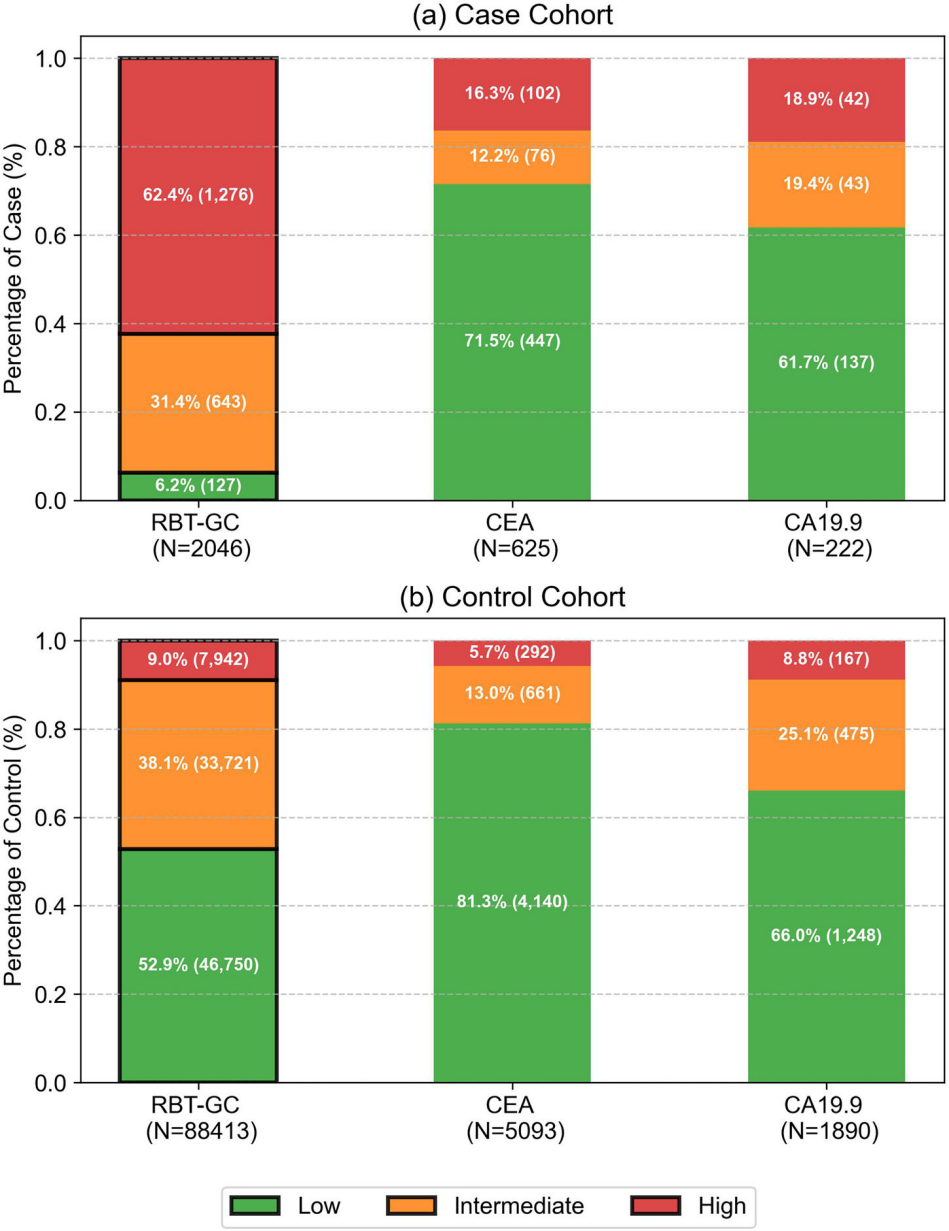
Comparison of RBT-GC biomarker and tumour marker levels in validation set. Comparison of biomarker risk group distributions between **a** GC case cohort and **b** control cohort in validation set. The cutoff of 0.01 and 0.06 were used to classify cohorts into high, intermediate and low risk. Each stacked bar represents the percentage and count of participants classified into low (green), intermediate (orange) and high (red) risk groups based on three biomarkers. Higher proportions of GC cases fall into the high risk category for RBT-GC (62.4%), whereas the control cohort shows predominance of low risk classifications across all biomarkers. This demonstrates RBT-GC’s ability to identify patients requiring urgent endoscopic evaluation.

The performance of the biomarker at two cutoffs was compared to that of CEA and CA19.9 (Table 2, Fig. 4, Supplementary Table 2). GC prevalence increases with risk scores. The prevalence reached 13.8% in the high-risk validation cohort, which is approximately six times the baseline of 2.3%. RBT-GC biomarker stratified 1276 cases as high-risk group, compared to 102 by CEA and 42 by CA19.9. RBT-GC biomarker yielded a high Negative Predictive Values (NPV) of 99.7% (46,750/46,897) in the low-risk group, which substantially surpassed that of CEA (90.3%; 4140/4587) and CA19.9 (90.1%; 1248/1385). RBT-GC biomarker identified fewer GC cases as low risk (*n* = 147) with a lower percentage (7.1%) compared to CEA (*n* = 447; 71.5%) and CA19.9 (*n* = 137; 61.7%). These findings indicate that RBT-GC achieves a high NPV, indicating that a low-risk classification reliably excludes GC.

**Fig. 5:**
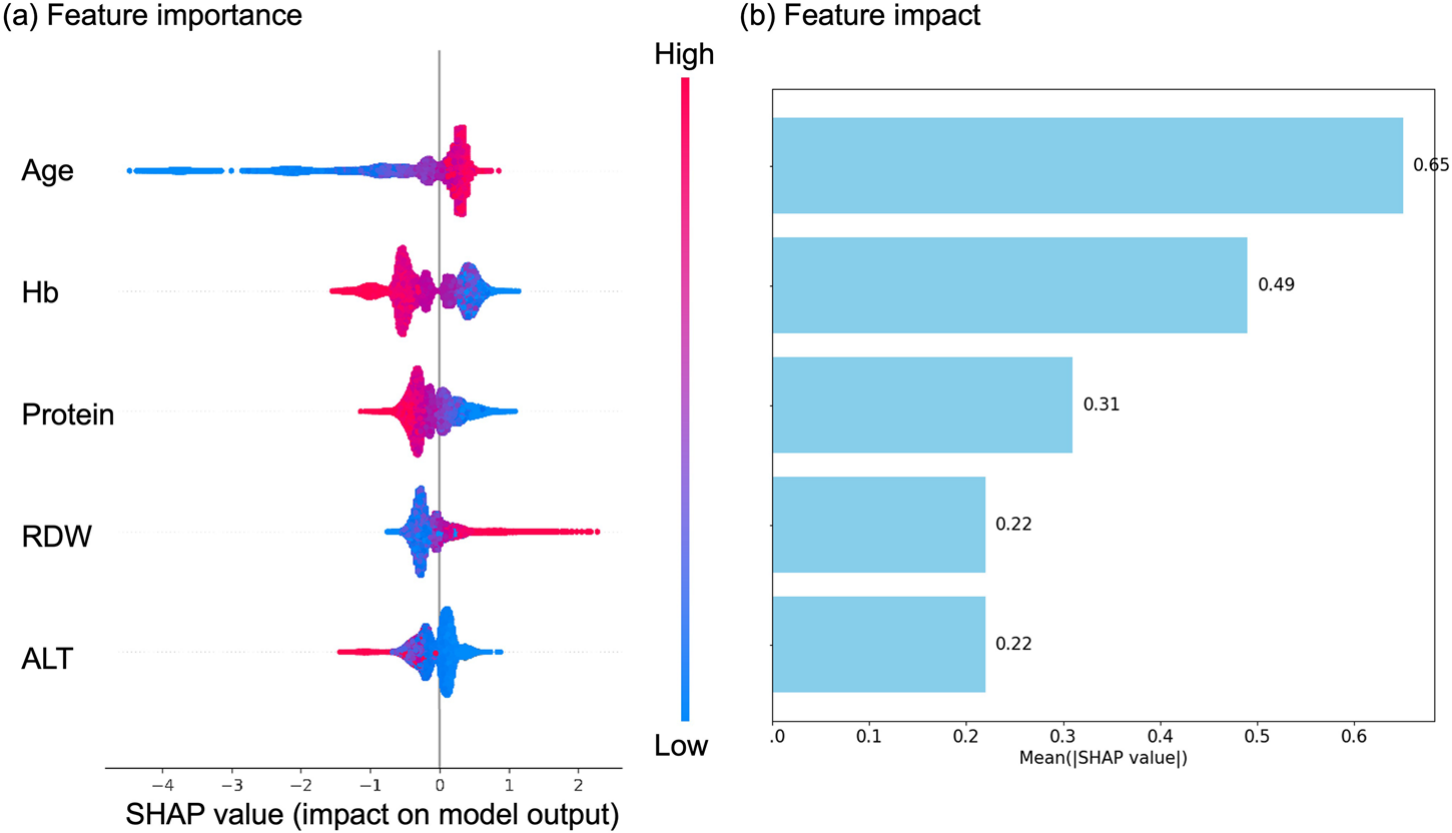
RBT-GC biomarker’s importance and impact in validation set. **a** Feature importance: SHAP summary plot showing how each feature contributes to the model’s output. Features are ranked by importance from top to bottom, with colour indicating feature value (red means high and blue means low). Age, Hb and protein have the highest influence on model predictions, aligned with Fig. 2. **b** Feature impact: Bar plot of mean absolute SHAP values representing each feature’s overall impact on the model. Age (0.65) shows the greatest effect, followed by Hb (0.49), protein (0.40).

The Beeswarm plot (Top 5 important features in Fig. 5, and all features in Supplementary Fig. 3) illustrates the relative importance of features driving RBT-GC’s risk assessment. The plot indicates that older age, lower Hb, lower protein level, higher RDW, and lower alanine aminotrasnferase (ALT) have a high positive impact on the prediction. The model identifies older age, more severe anaemic condition (low Hb, and high RDW) and more malnutritional status (low protein, and low ALT) as key decision-making factors.

### Comparison to other risk indices

The performance of RBT-GC was the most accurate among all studies that used patient cohort with chronic dyspepsia and similar case-control ratio (Table 3). The accuracy of the model surpassed AUROC of 0.75 reported by Read et al.^20^ and of 0.71 reported by Park et al.^21^. The number of cases and controls in our study represents the largest patient cohort to date, implying our findings are robust and generalisable.

**Table 3:** Comparison of RBT-GC biomarker to selected GC risk score system

Citation	Features used	Cohort	Control	Case	AUROC
Questionnaires					
Leung et al.^13^	Demographic, comorbidity, medications	Patients who received HP eradication therapy	89,349	189	0.97
Zhou et al.^34^	Demographic, diet, lifestyle	(1) Age 45–69 years, (2) no cancer history, (3) upper GI endoscopy	47,954	125	0.76
Gohari et al.^35^	Demographic, diet, medical status, lifestyle	Non-ulcer dyspeptic patients	1132	858	0.86
Park et al.^21^	Demographic, medical status, lifestyle	(1) Age 40–74 years, (2) gastroscopy	10,450,292	65,657	0.71
Serological tests and molecular biomarkers					
Liang et al.^36^	Demographic, CEA, CA19.9, CA72.4	Healthy or with benign gastric diseases	1869	2288	0.63
Yu and Zheng^37^	CEA, CA19.9, CA72.4	Benign gastric/colorectal disease	49	216	0.77
Gong and Zhang^26^	CEA, CA19.9, CA72.4, anti-Hp antibody	GC-positive cases, GC-negative controls	204	206	0.92
Zhou et al.^38^	Demographic, serological test (HP-igG, PG I/II ratio, G-17)	(1) Digestive symptoms or health examinations, (2) Gastroscopy	6300	401	0.79
So et al.^15^	12-miRNA assay, age, anti-HP IgG + CEA, CA19.9, PGI/II	GC-positive cases, GC-negative controls	236	236	0.89
Routine blood test					
Taninaga et al.^27^	Demographic, comorbidity, CBC, HP test	Healthy subjects	1342	89	0.90
Read et al.^20^	Demographic, CBC, BMP	Patients with 2 CBCs within 2 year	148,069	1025	0.75
This study	Demographic, CBC, LRFT	Chronic dyspeptic patients	347,650	5918	0.87

## Discussion

GC has higher mortality in regions without population-based GC screening, suggesting the importance in implementing a pragmatic and affordable cancer screening programme. Cost-effectiveness is particularly an important agenda for countries with the medium-high GC incidence as many of them are low-to-middle income countries (LMIC)^22,23^. RBT-GC repurposes RBT for triage before endoscopy and mitigates the issue of high cost associated with endoscopic screening. Affordable screening and triage methods can improve the risk-benefit balance and programme cost, which potentially enables screening work in LMICs.

In our previous study, we conducted a comprehensive analysis of RBT components to differentiate GC cases from controls. All 24 components were significantly different (*p* < 0.05) between cases and controls. Multivariate logistic regression (LR) identified components indicating blood loss, red cell turnover, systemic inflammation, and liver involvement are valuable for distinguishing GC cases. Our previous paper established the clinical foundation for RBT to be used for GC detection. However, statistical modelling such as LR, has an inherent weakness in modelling dataset with significant interactions between parameters. This issue can be illustrated in complete blood count (CBC). Clinically, patients with anaemia have low haemoglobin and high platelet count simultaneously, while mean corpuscular haemoglobin (MCH), mean corpuscular volume (MCV), mean corpuascular haemoglobin concentration (MCHC), and RDW could be altered in different ways depending on the aetiology^24^. Building LR models portends the choice of a few components among the many to reduce collinearity during model building. In contrast, machine learning algorithms allow inclusion of all parameters during model building without compromising its classification ability without selection.

Our evaluation of six machine learning algorithms revealed that XGB (the leftmost column in Fig. 2) achieved the highest classification accuracy. The top 10 features identified by SHAP values were consistent across XGB, light gradient boosting machine (LGBM), gradient boosting (GB), adaptive boosting (ADA) and random forest (RF) models. The performance of these models was comparable in terms of classification accuracy and SHAP feature rankings. These findings suggest that the GC biomarker within the current dataset is robust and independent of the specific machine learning algorithm employed. This highlights the potential of machine learning to reliably identify clinically relevant biomarker for risk triage before OGD. LR (the rightmost column in Fig. 2) identified a distinct feature set compared to other models in SHAP analysis. The LR SHAP was consistent with a high correlation between RBC and Hb (*r* > 0.78) from a prior study^17^. Hb is a relevant diagnostic indicator for anaemia and recognised to be more clinically significant than RBC. This discrepancy highlights the limitations of traditional linear models in capturing complex GC patterns in high-dimensional RBT data.

The primary strength of the RBT-GC model lies in its ability to optimise the triage process for OGD. Current GC screening relies heavily on OGD with an NNS of 40–70 (from HADCL). RBT-GC identifies high-risk patients for prioritised OGD. This reduces the NNS to 15 in high-risk group, significantly improving the pick-up rate of GC. DCA confirmed that the triage using RBT-GC model offers higher net benefit than treating all or none within the selected threshold range. Furthermore, RBT-GC model provides explainable predictions aligned with clinical expectations (Fig. 5). SHAP analysis indicated that older subject age, signs of anaemia (low Hb, higher RDW) and indicators of malnutrition (low protein and ALT levels) increase RBT-GC risk scores. This enables clinicians to link clinical observations and predict GC risks without additional resources. The HADCL database in 2000–2020 showed RBT was used in 57% (5198/10,283) of cases, compared to 18% (1928/10,283) for CEA and 6% (645/10,283) for CA19.9. RBT-GC classified 61.8% of cases as high-risk, whereas tumour markers (TMs) identified less <60% of cases. RBT-GC can identify patients requiring urgent endoscopic evaluations. The opportunistic use of RBT-GC enables near-diagnosis triage as it can be used in undiagnosed or unsuspected subjects, where TMs are used mainly in highly suspected subjects.

The clinical implementation potential of our approach is strengthened by the ubiquitous nature of the underlying data. RBT is routinely used to assess general health without requiring disease-specific justification. UK guidelines recommend a full blood count to screen dyspeptic individuals^25^ while European guidelines suggest RBT on GC high-risk groups^4^. Our large cohort (347,650 controls and 5918 cases) reflects the frequent usage. Panels such as CBC, liver function test (LFT) and renal function test (RFT) are widely accessible and easier to collect than TMs, gastrointestinal biomarkers, or detailed disease histories. This reliance on standard RBT makes RBT-GC biomarker more practical to implement than indices requiring specialised biomarkers or extensive clinical data. RBT-GC biomarker imposes minimal clinical burden.

RBT-GC biomarker remains robust even with limited data. Although the primary model required 19 of 24 components, validation results showed that accuracy remained high (AUROC > 0.75) even when using smaller subsets of data. A model trained on CBC components alone is shown to perform as well as the full-panel model. In case of missing blood component values, the panel-specific sub models will ensure flexibility and accuracy.

In performance comparisons with existing approaches, the present model demonstrates favourable characteristics. The AUROC of 0.87 for our model (the bottom row in Table 3) suggests a high accuracy for identifying GC within a large population of chronic dyspepsia. While some studies reported higher AUROCs, their findings are not directly comparable due to differences in study design and cohort volume. Leung et al.^13^ focused on a highly specific cohort of patients with HP treatment, which may limit the generalisability to broader population. Findings from Gong and Zhang^26^ and Taninaga et al.^27^ are based on small sample sizes, which makes their results less robust. Park et al.^21^ examined a broader population who underwent gastroscopy at age of 40–74 in Korea, but the model is based on demographic and clinical features only and cannot be directly compared. Our study cohort encompasses a territory-wide 347,650 controls and 5918 cases, representing the chronic dyspeptic population which is the main target for endoscopy in clinical practice. The size of the dataset ensures a more robust evaluation of triage than previous studies.

Several limitations of this study warrant consideration. Regarding model architecture, a conservative scale_pos_weight was adopted to prioritise calibration over addressing the underlying class imbalance. Future research may investigate post-hoc calibration methods to address the class imbalance. Selection bias may exist due to the retrospective nature of the study. The comparison between RBT-GC and TMs was restricted to patients who underwent both tests. Since TMs are typically ordered for high-risk individuals, excluding subjects without TM results may have introduced indication bias. This limitation could be mitigated by simultaneous testing in future prospective studies.

The current study developed and validated the RBT-GC biomarker in a territory-wide, 20 year clinical cohort which accurately detects GC. The biomarker is highly practical and scalable, and holds great potential of enabling affordable GC triage by prioritising high-risk patients for further diagnostic workup including OGD and imaging.

## Methods

### Data source

This is a territory-wide retrospective study (HREP-2022-0346 and HREP-2024-0004 by Human and Artefacts Research Ethics Committee at the Hong Kong University of Science and Technology and KC/KE-23-0013/ER-2 by Research Ethics Committee at Kowloon Central/Kowloon East Cluster in Hong Kong Hospital Authority) with no formal patient and public involvement. This study used de-identified data from the Hong Kong Hospital Authority Data Collaboration Lab (HADCL) where the requirement of informed consent was waived. The database holds anonymised data of over 3 million patients from Hong Kong public hospitals and clinics. The data includes information including medical records, diagnosis, medication, radiology results, laboratory test results from 2000 to 2020.

### Cohort definition

All patients older than 18 and with prescribed medication for dyspepsia (Supplementary Table 5) were retrieved. Medication prescription history was used to ensure the cohort truly reflects chronic dyspepsia, as diagnosis codes alone often lack the specificity to accurately identify the targeted chronic population. A dual-confirmation protocol of using International Classification of Disease, Tenth Revision, Clinical Modification (ICD-10-CM) codes and oesgophagogastroduodenoscopy-based pathological reports were used to identify GC cases. GC cases were dyspeptic patients with primary GC diagnosed between January 1, 2000, to December 31, 2020. Controls were dyspeptic patients without the records and history of cancer. Both cases and controls were free from simultaneous tumours at organs other than stomach.

RBT record included CBC, LFT and RFT (Supplementary Table 6). They were selected as predictors due to their routine availability^4^ and established associations with GC in literature^16,17,28,29^. One RBT record per subject was collected from a period within 1 month before the GC diagnosis for the cases, and 1 month after the dyspeptic medication for the controls. A 1-month window was chosen to capture RBT data close to GC diagnosis or dyspepsia onset. Only the subjects with RBT records within the specified window and at least 19 of 24 (80%) components were included. Selection bias is examined by analysing model performance within smaller sub-panels. Each blood records were statistically analysed using Student’s *t* test and normalised using its upper and lower limit with the following equation:

$${Normalised}\,{result}=\,0.20+\,\frac{(0.80-0.20)}{({Upper}\,{limit}-{Lower}\,{limit})}$$

Eligible patients were divided into training, testing and validation cohort based on diagnosis year. Data from 2000 to 2015 was allocated for training (207,392 controls and 3071 cases) and testing (51,845 controls and 781 cases) with the ratio of 8:2. Data collection for the training and testing cohort was extended to 2020, where controls were required to have a minimum of 5 years of follow-up without any indication of cancer. Data from 2016 to 2020 were reserved for validation (88,413 controls and 2066 cases). As no data were available beyond 2020, the follow-up period for validation set was inherently limited. Each patient contributed only one record to the dataset, and data division was performed at the patient level to avoid any overlap between the subsets. Training cohort was used to develop a model, testing cohort was used to examine the classification performance of various models, and validation cohort was used to validate the clinical application of the selected model. The homogeneity of the large-scale dataset has the advantage for initial model development as it minimised genetic and environmental confoundment, allowing the model to learn in a less noisy environment. Validation in other territories will be conducted after the model is established. The study protocol is neither publicly available, nor registered as the data was originally collected by HADCL for other purposes.

### Model development and evaluation

Six machine learning models including Logistic Regression, Random Forest, Gradient Boosting, Light Gradient Boosting Machine, Extreme Gradient Boost, Adaptive Boosting, Decision Tree were used to develop RBT-based models using 24 RBT components and two demographic features. Model calibration was assessed using calibration plots and quantitative metrics of slope and intercept. During model selection, missing values were imputed using the median of the training set. The model was used to calculate GC risk scores (0–1). Shapley additive explanations (SHAP) values were calculated to quantify the contribution of each training parameter to the GC risk prediction by each model. XGB was selected based on SHAP feature rankings, AUROC, AUPRC, a stability in calibration plots and the ability to handle missing data.

For the final model development, XGB was trained directly on the dataset with missing values to maintain clinical applicability. The hyperparameter configurations used were scale_pos_weight of 2, n_estimators of 500, max_depth of 4, learning rate of 0.05 and gamma of 0.4. In real-world clinical settings, not all patients undergo comprehensive testing for all components of CBC, LFT and RFT during a single visit. This results in naturally occurring missing data, which reflects the variability and incompleteness inherent in routine medical practice. No hospital-level clustering was modelled, as HADCL data were treated as a single cohort.

Predictions were generated using a XGB model in Python 3.7.6, with scores output as probabilities from 0 to 1. Two thresholds were determined using independent testing sets. A lower cutoff of 0.01 (90% sensitivity) and an upper cutoff of 0.06 (90% specificity) were used to divide the cohort into low-risk (0–0.01), intermediate risk (0.01–0.06) and high risk (0.06–1.0) groups. Thresholds were fixed and applied to the validation set. Model performance was evaluated using Receiver Operating Characteristic curves, Precision-Recall Curves, calibration plots and DCA, while SHAP plots were utilised for interpretability. Primary metrics included discrimination (AUROC and AUPRC) and classification indices (sensitivity and specificity). Secondary utility indices included Positive and Negative Predictive Values (PPV, NPV) and the NNS, defined as the total patients within a stratum divided by detected GC cases. Subgroup analyses based on biological sex and age were also conducted.

### Comparison to traditional tumour markers

RBT-GC biomarker was compared to CEAand CA19.9 in terms of data volume, classification accuracy and implementation feasibility. No imputation was performed for missing TM data. Per-marker analysis was conducted to reflect the real-world diagnostic accuracy of each test in the population where it is currently utilised. We assessed the classification performance in terms of sensitivity, specificity, implementation feasibility and NNS. The two cutoffs selected were 5 ng/mL and 10 ng/mL for CEA, and 37 U/mL and 100 U/mL for CA19.9. The lower thresholds (CEA 5 ng/mL; CA19.9 37 U/mL) are universally accepted for GC^30,31^. The higher thresholds (CEA 10 ng/mL^32^; CA19.9 100U/mL^33^) are frequently used in clinical practice to define high risk.

## Supplementary information

Supplementary information

## Data Availability

The datasets generated and/or analysed during the current study are not publicly available due to confidentiality restrictions mandated by the data provider in accordance to Hong Kong Laws, but are available from the corresponding authors on reasonable request.
