# Supplementary material for: Development and validation of a novel blood-based biomarker for gastric cancer triage in chronic dyspepsia
Source: NPJ Digit Med. 2026 Apr 17;9:466. doi: 10.1038/s41746-026-02618-1 (PMC13273075; doi:10.1038/s41746-026-02618-1)
Supplement: Supplementary file 1 — Supplementary information [file 41746_2026_2618_MOESM1_ESM.docx]

**Supplementary Figure 1. Performance of all machine learning models on the test set.** (a) Receiver operating characteristic curves for six machine learning models, XGB, Extreme gradient boosting; GB, gradient boost; LGBM, light gradient boosting machine; ADA, adaptive boost; RF, random forest; LR, logistic regression.


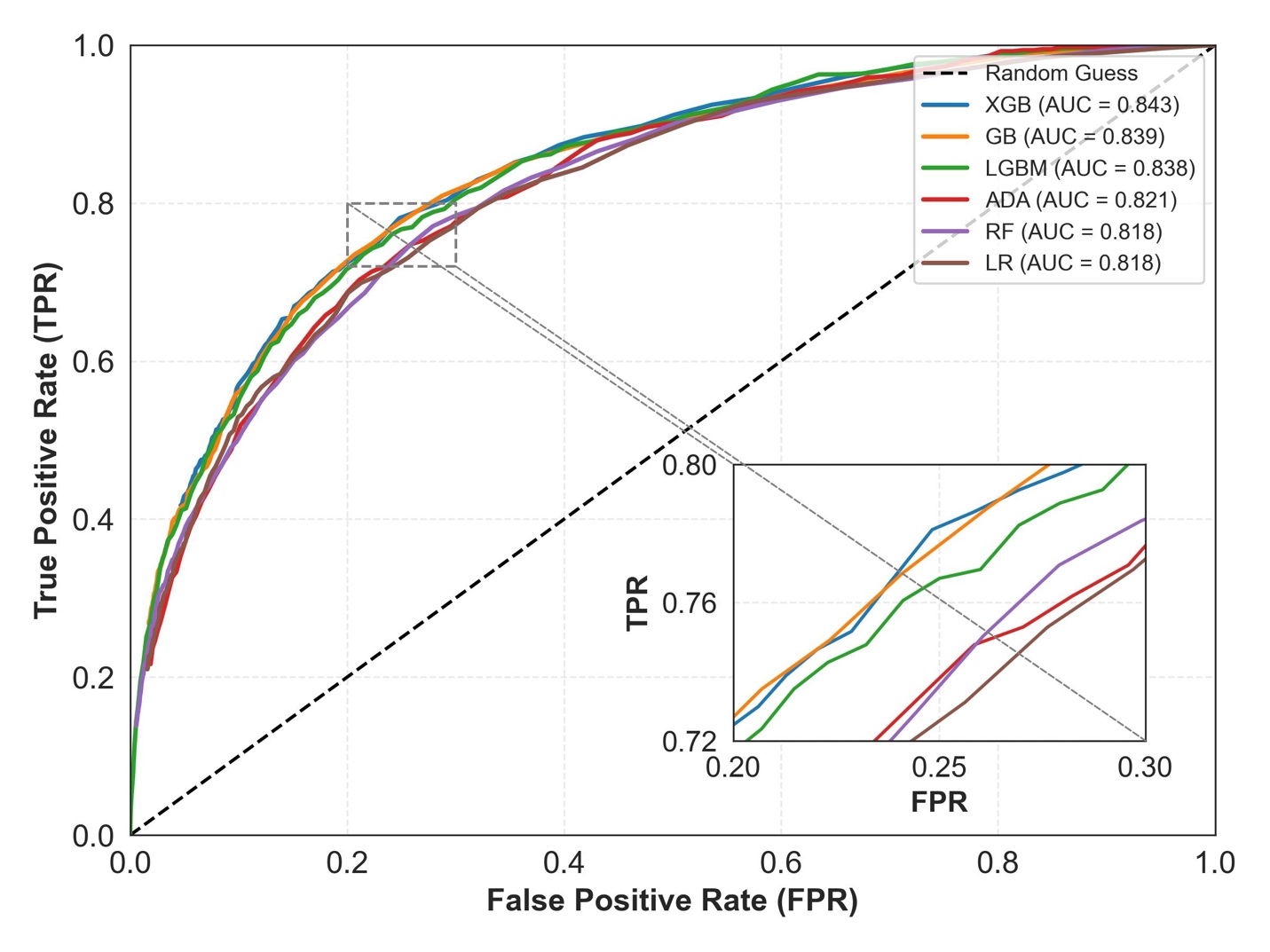


(b) Precision-recall curves for the same six models, and


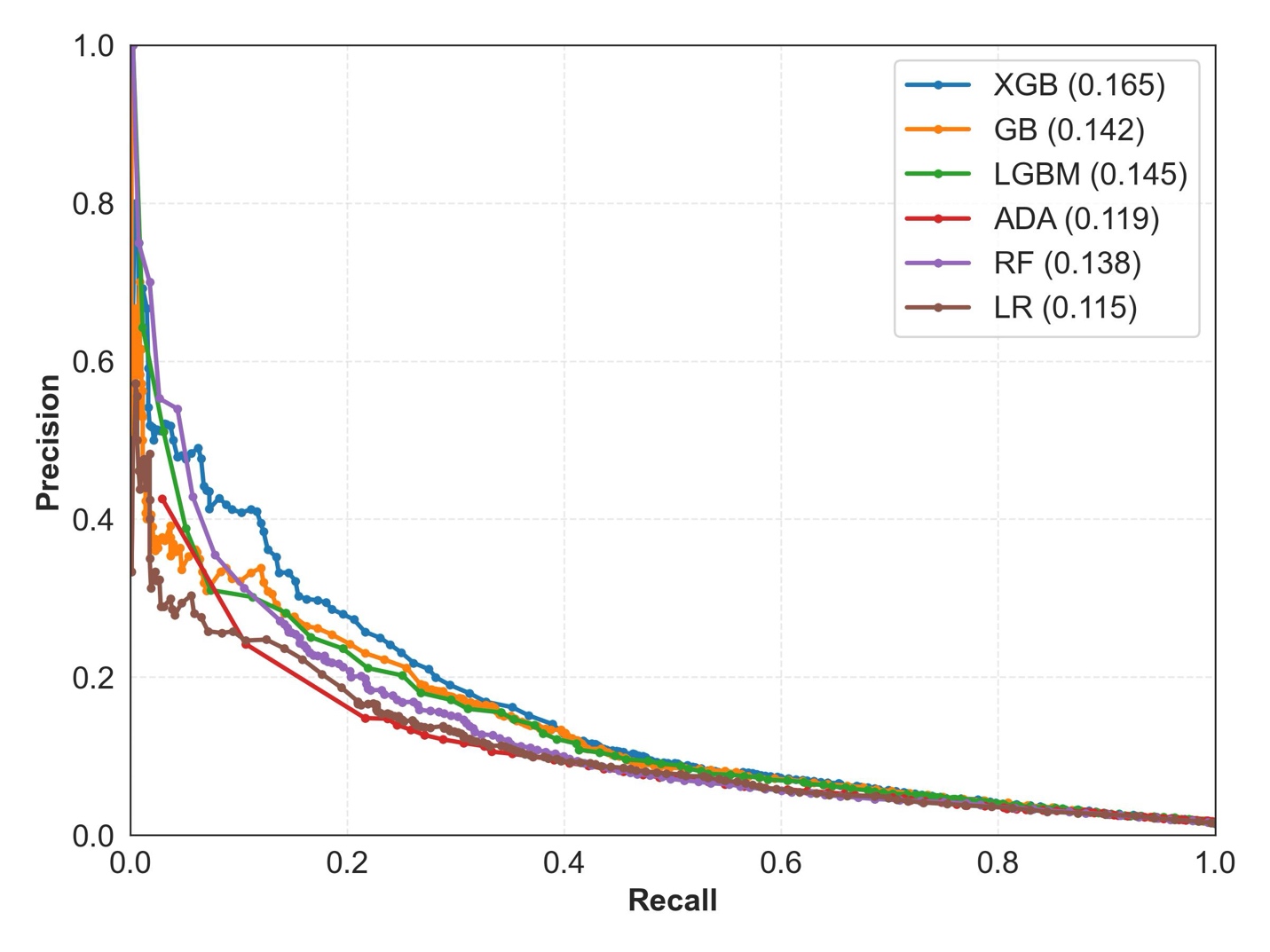


(c) Calibration curve for the same six models,


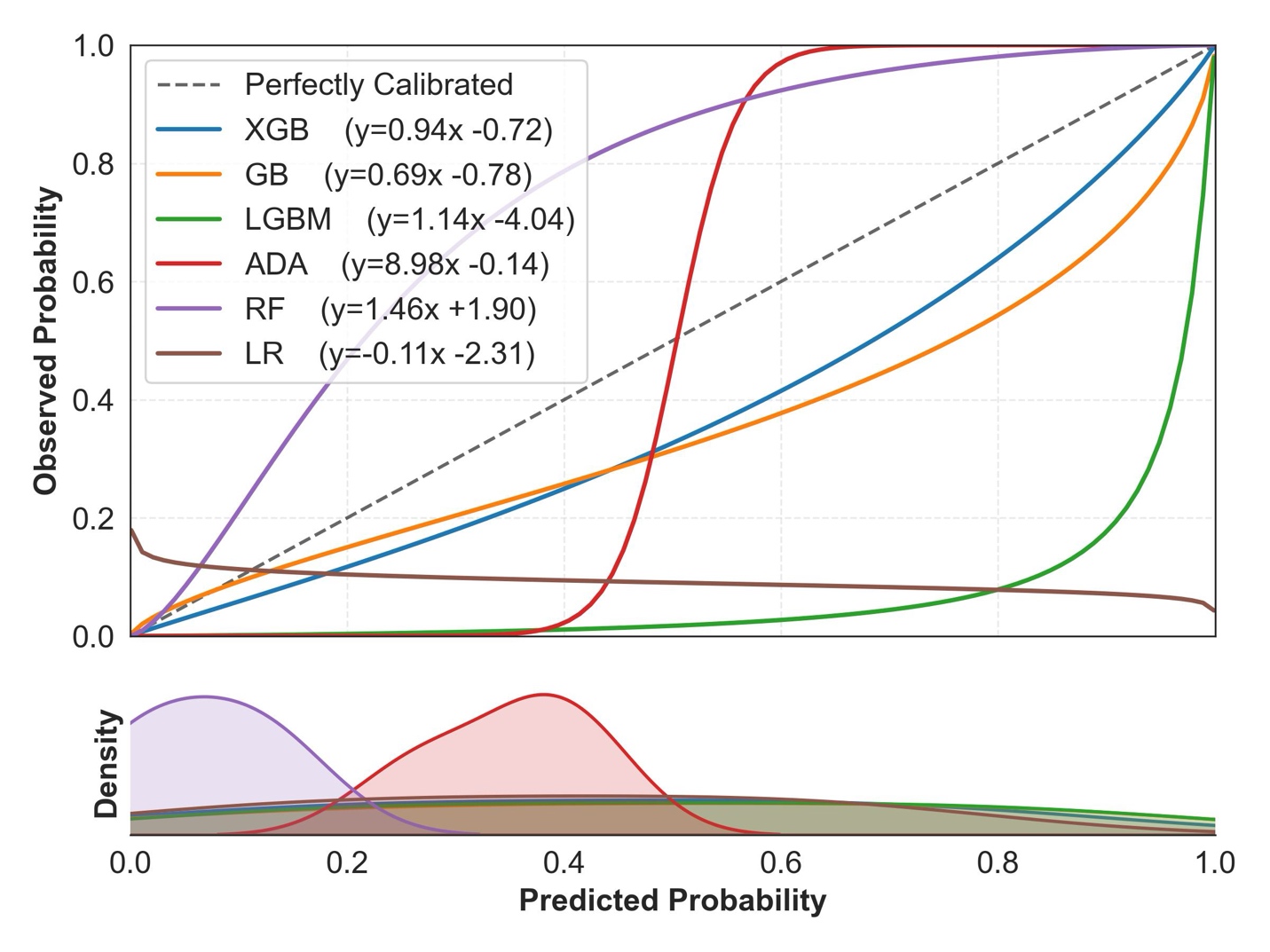


**Supplementary Figure 2. Performance of the XGBoost model across training and validation sets.** (a) Receiver operating characteristic (ROC) curve for the RBT-GC model,


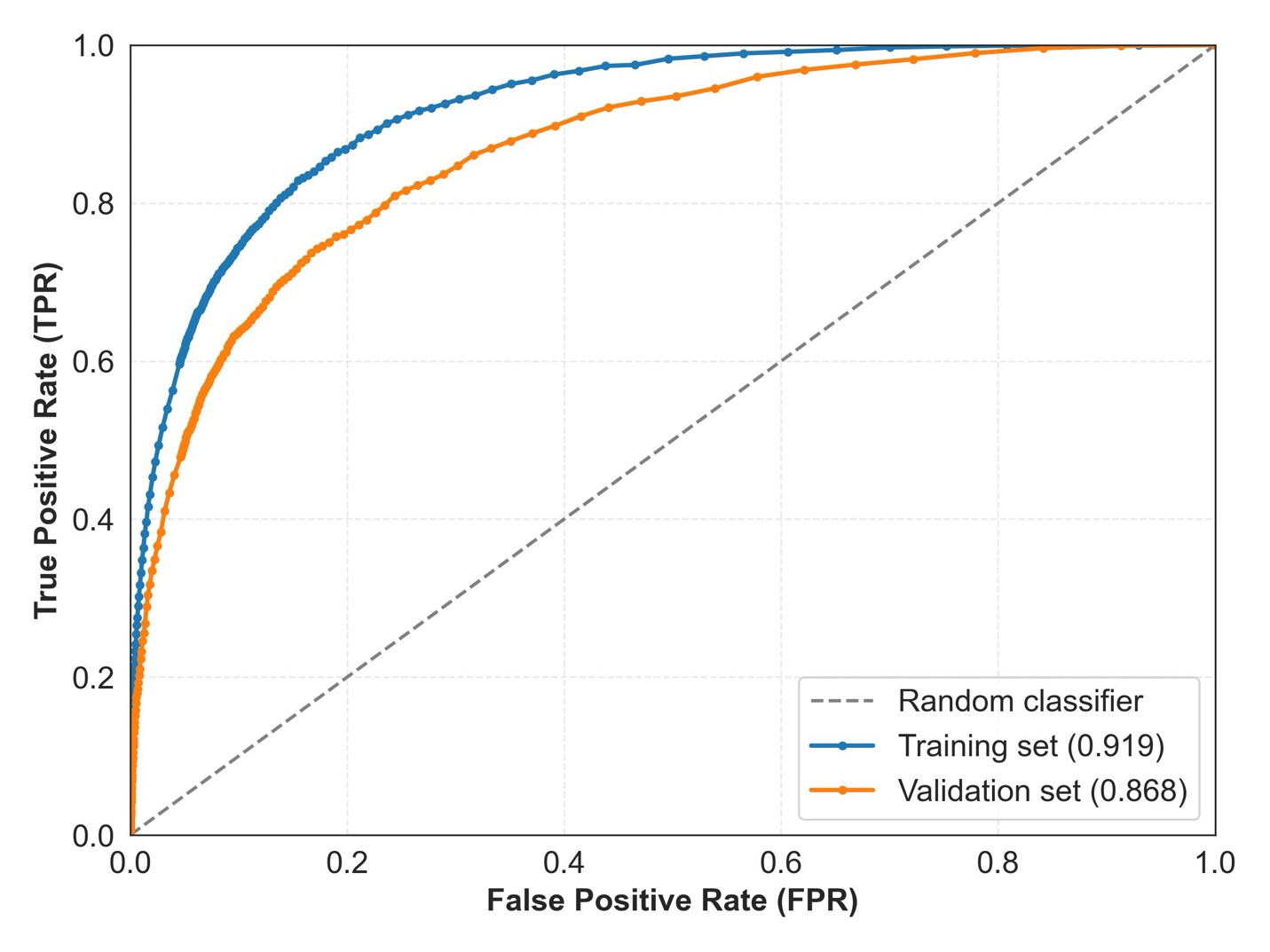


(b) Precision-recall curve for the RBT-GC model,


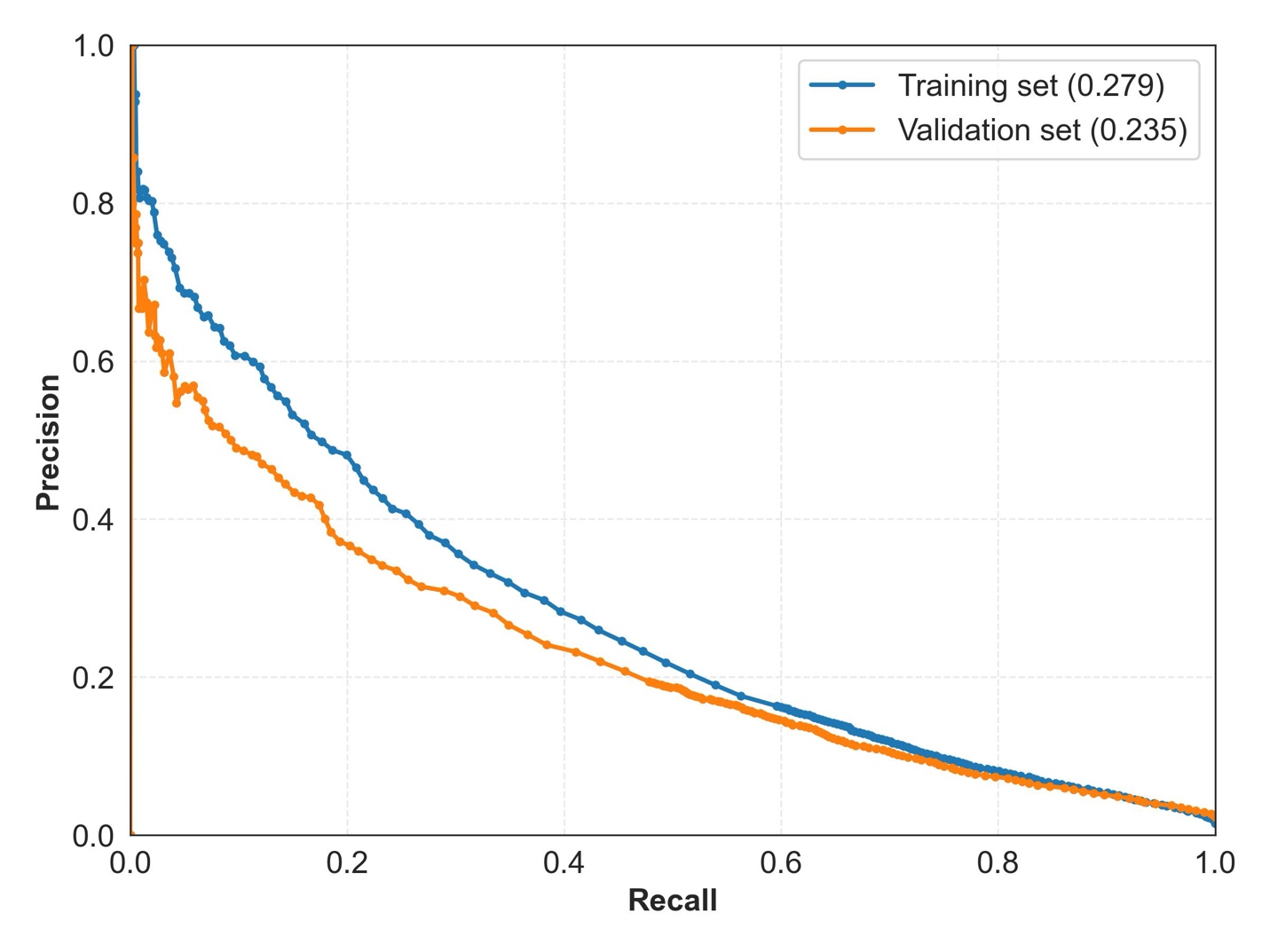


(c) Calibration curve for the RBT-GC model,

The calibration curve for the validation cohort is shown in Supplementary Figure 5c. The model is well-calibrated with a calibration slope of 1.01 (95% CI, 0.91-1.11) and an intercept of -0.15 (95% CI, -0.32-0.02).


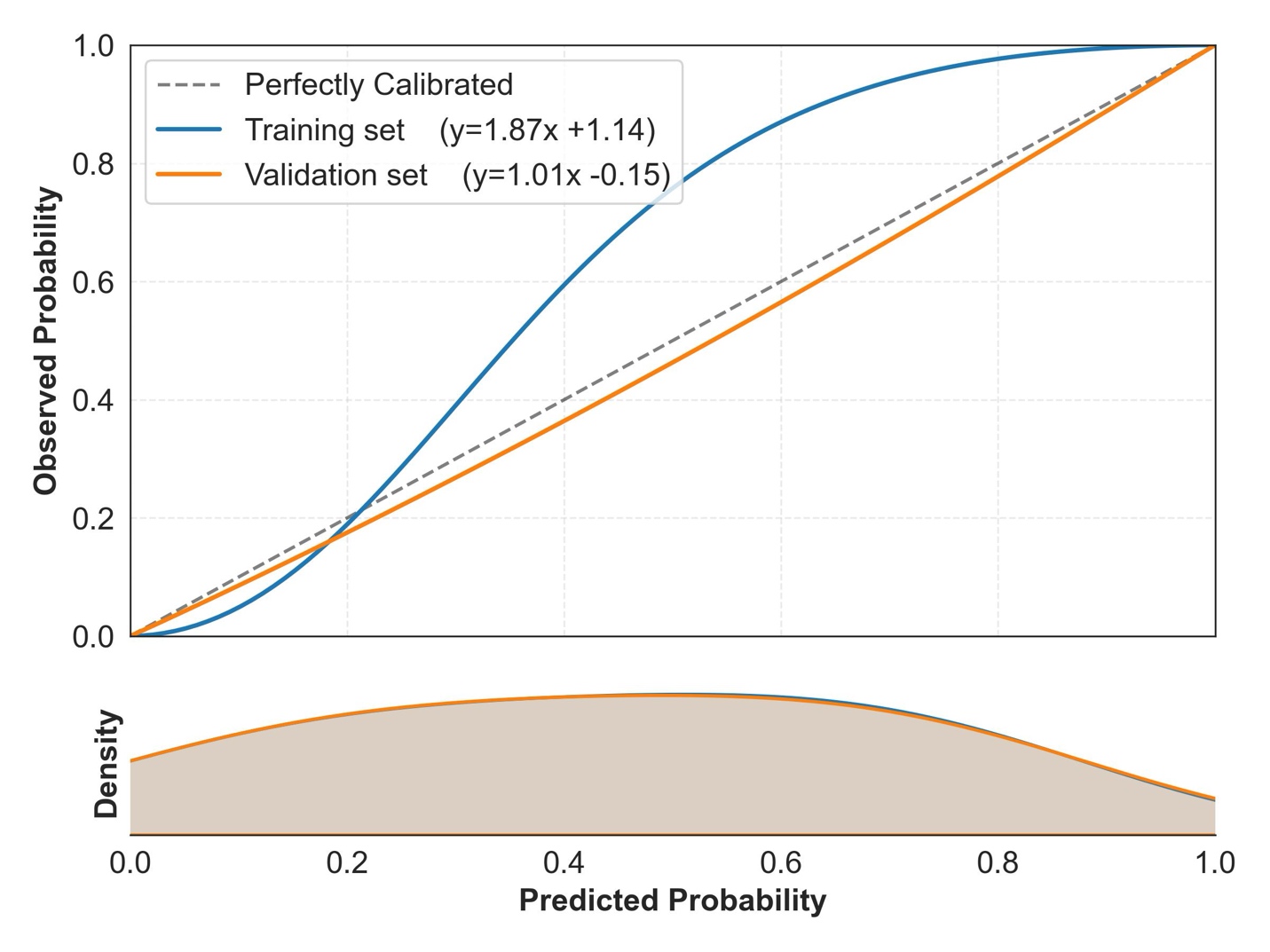


(d) Decision curve analysis for the RBT-GC model


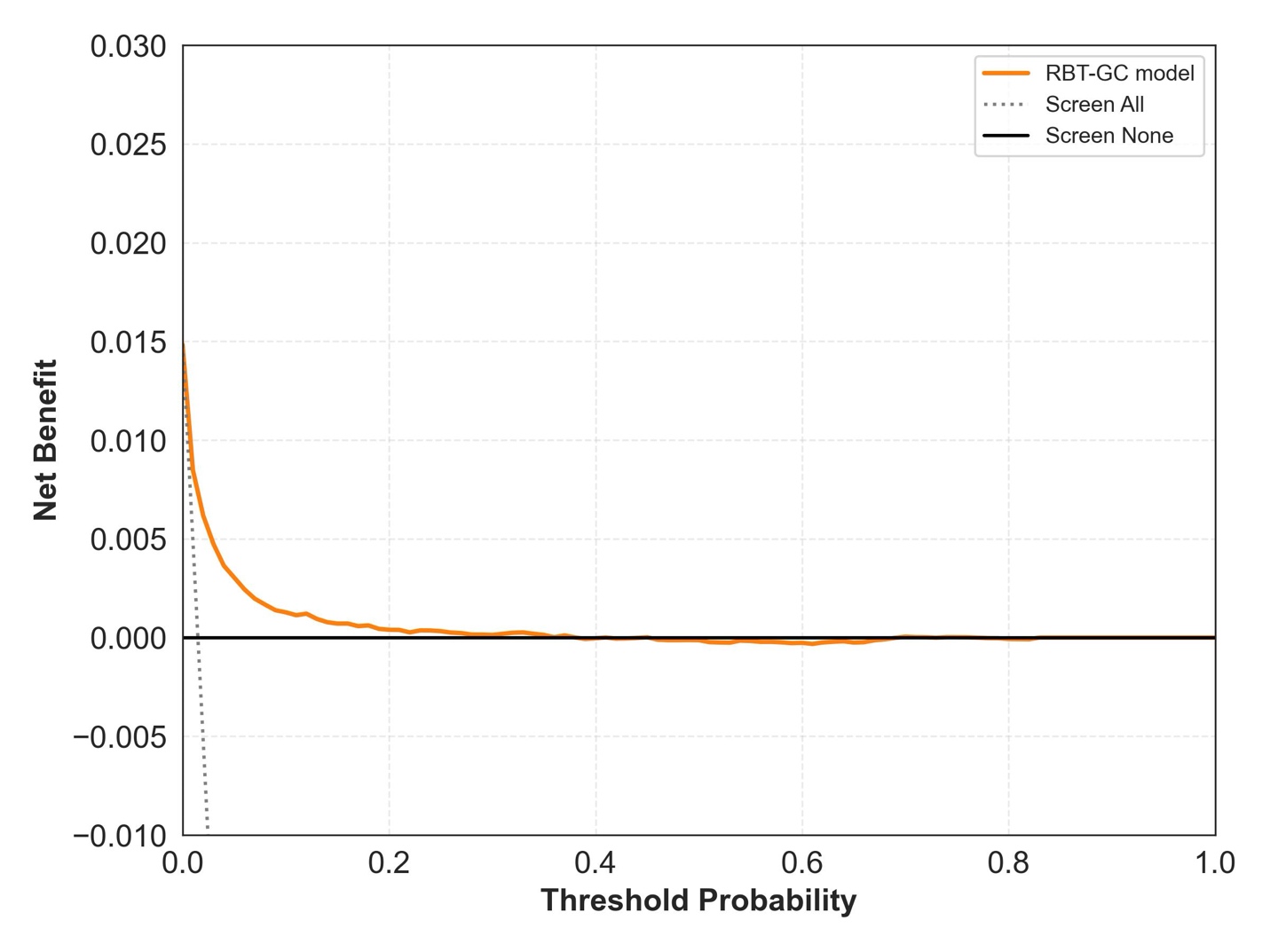


Supplementary Figure 3. Full SHAP summary plot in validation set. SHAP, Shapely additive explanations; Hb, haemoglobin; RDW, red blood cell distribution width; ALT, alanine transaminase concentration; MCH, mean corpuscular haemoglobin; RBC, red blood cell; ALP, alkaline phosphatase concentration; MCV, mean corpuscular volume; WBC, white blood cell count; MCHC, mean corpuscular haemoglobin concentration.


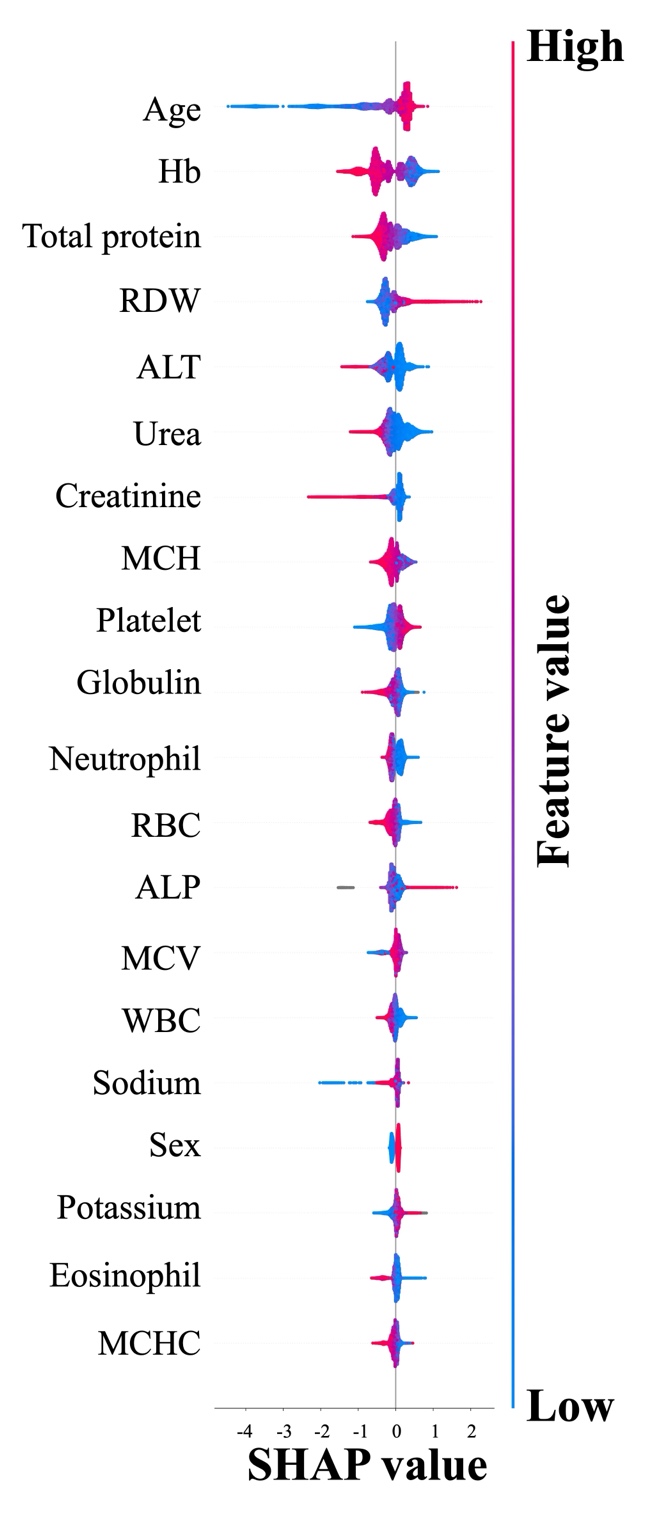


Supplementary Table 1. Incidence and mortality rate per 100,000 in Japan, South Korea, China, and other countries with moderate-to-high incidence rate in 2022. GC, gastric cancer; ASR, age-standardized ratio; M/I, mortality/incidence

| Country | ASR Incidence  (per 100,000) | ASR Mortality  (per 100,000) | M/I ratio | GC Screening Program Coverage |
| --- | --- | --- | --- | --- |
| Japan | 27.6 | 7.2 | 0.26 | Nation-wide |
| South Korea | 27.0 | 6.5 | 0.24 | Nation-wide |
| China | 13.7 | 9.4 | 0.68 | Regional |
| Eastern Europe | 11.1 | 7.8 | 0.70 | None or pilot programs |
| South America | 9.1 | 7.0 | 0.77 | None or pilot programs |

Source: IARC Global Cancer Observatory (Cancer Today)

Supplementary Table 2. Retrospective classification performance per cutoffs in (a) test set and (b) validation set.

RBT-GC, routine blood test – gastric cancer model; CEA, carcinoembryonic antigen; CA19.9, carbohydrate antigen 19.9, NNS, number needed to scope.

| Test set | RBT-GC index | CEA | CA19.9 |
| --- | --- | --- | --- |
| Controls (N) | 51,845 | 2,000 | 236 |
| Cases (N) | 781 | 185 | 80 |
| NNS | 66.4 | 10.8 | 3.0 |
| Using a lower cutoff | 0.01 | 5 ng/ml | 37 ng/ml |
| True negative (specificity, %) | 26,042 (50%) | 1,615 (81%) | 177 (75%) |
| True positive (sensitivity, %) | 707 (91%) | 60 (32%) | 26 (33%) |
| Missed GC, n/total (%) | 74 (9%) | 125 (68%) | 54 (67%) |
| NNS | 36.5 | 6.4 | 2.3 |
| Using an upper cutoff | 0.06 | 10 ng/ml | 100 ng/ml |
| True negative (specificity, %) | 47,039 (91%) | 1,899 (95%) | 207 (88%) |
| True positive (sensitivity, %) | 435 (56%) | 40 (22%) | 16 (20%) |
| Missed GC, n/total (%) | 346 (44%) | 145 (78%) | 64 (80%) |
| NNS | 11.0 | 2.5 | 1.8 |

| Validation set | RBT-GC index | CEA | CA19.9 |
| --- | --- | --- | --- |
| Controls (N) | 88,413 | 5,093 | 1,890 |
| Cases (N) | 2,066 | 625 | 222 |
| NNS | 42.8 | 8.1 | 8.5 |
| Using a lower cutoff | 0.01 | 5 ng/ml | 37 ng/ml |
| True negative (specificity, %) | 46,750 (53%) | 4,140 (81%) | 1,514 (80%) |
| True positive (sensitivity, %) | 1,919 (93%) | 178 (28%) | 59 (27%) |
| Missed GC, n/total (%) | 147 (7%) | 447 (72%) | 163 (73%) |
| NNS | 22.7 | 6.3 | 7.4 |
| Using an upper cutoff | 0.06 | 10 ng/ml | 100 ng/ml |
| True negative (specificity, %) | 80,471 (91%) | 4,801 (94%) | 1,723 (91%) |

Supplementary Table 3. Classification performance in validation set in (a) age subgroups, (b) biological sex subgroups.

| Age | Age < 50 | 50’s | 60’s | Age >= 70 |
| --- | --- | --- | --- | --- |
| Controls (N) | 27,398 | 17,591 | 17,959 | 25,465 |
| Cases (N) | 100 | 214 | 462 | 1,290 |
| NNS | 274.0 | 82.2 | 38.9 | 19.7 |
| Using a lower cutoff | 0.01 | 0.01 | 0.01 | 0.01 |
| True negative (specificity, %) | 22,823 (83%) | 9,911 (56%) | 7,197 (40%) | 6,819 (27%) |
| True positive (sensitivity, %) | 68 (68%) | 179 (84%) | 422 (91%) | 1,250 (97%) |
| Missed GC, n/total (%) | 32 (32%) | 35 (16%) | 40 (9%) | 40 (3%) |
| NNS | 68.2 | 43.9 | 26.5 | 16.0 |
| Using an upper cutoff | 0.06 | 0.06 | 0.06 | 0.06 |
| True negative (specificity, %) | 27,132 (99%) | 16,638 (95%) | 16,174 (90%) | 20,527 (81%) |
| True positive (sensitivity, %) | 20 (20%) | 90 (42%) | 242 (52%) | 924 (72%) |
| Missed GC, n/total (%) | 80 (80%) | 124 (58%) | 220 (48%) | 366 (28%) |
| NNS | 14.3 | 11.6 | 8.4 | 6.3 |

(b) biological sex subgroups

| Biological sex | Male | Female |
| --- | --- | --- |
| Controls (N) | 51,665 | 36,748 |
| Cases (N) | 1,283 | 783 |
| NNS | 40.3 | 46.8 |
| Using a lower cutoff | 0.01 | 0.01 |
| True negative (specificity, %) | 25,667 (50%) | 21,083 (57%) |
| True positive (sensitivity, %) | 1,216 (95%) | 703 (90%) |
| Missed GC, n/total (%) | 67 (5%) | 80 (10%) |
| NNS | 22.4 | 23.3 |
| Using an upper cutoff | 0.06 | 0.06 |
| True negative (specificity, %) | 46,413 (90%) | 34,058 (93%) |
| True positive (sensitivity, %) | 847 (66%) | 429 (55%) |
| Missed GC, n/total (%) | 436 (34%) | 354 (45%) |
| NNS | 7.2 | 7.3 |

Supplementary Table 4. AUROC of RBT-GC model with limited features. CBC, complete blood count; LFT, liver function test; RFT, renal function test; WBC, white blood cell.

| Features | Training set | Testing set | Validation set |
| --- | --- | --- | --- |
| Demographics + CBC full + LFT + RFT | 0.92 | 0.84 | 0.87 |
| Demographics + CBC full | 0.88 | 0.81 | 0.84 |
| Demographics + CBC without WBC differential | 0.88 | 0.80 | 0.84 |
| Demographics + LFT | 0.84 | 0.78 | 0.79 |
| Demographics + RFT | 0.80 | 0.72 | 0.75 |

Supplementary Table 5. The list of chronic dyspeptic medications used

| Generic name | Trade name | Strength (mg) |
| --- | --- | --- |
| Aluminium hydroxide | Alusorb | 500 |
|  | Alutab | 600 |
| Aluminium hydroxide/ magnesium carbonate | Babcon | 20mg/mL |
| Aluminium hydroxide/ magnesium carbonate/ simeticone | Gasteel | 40 |
|  | Mylanta | N/A |
| Magnesium hydroxide | Milk of Magnesia | 7-8.5% W/W |
| Dexlansoprazole | Dexilant | 30 |
|  |  | 60 |
| Esomeprazole (magnesium trihydrate) | Nexium | 10/sachet |
|  |  | 20 |
|  |  | 40 |
| Famotidine | Pepcidine | 20 |
|  |  | 40 |
| Pantoprazole (sodium sesquihydrate) | Pantoloc | 20 |
|  |  | 40 |
| Rabeprazole sodium | Pariet | 10 |
|  |  | 20 |

Supplementary Table 6. The list of routine blood test parameters used and their units

| Blood functional group | Components | Unit |
| --- | --- | --- |
| Complete blood count | Red blood cell, count (RBC) | 10^9^/L |
|  | White blood cell, count (WBC) | 10^9^/L |
|  | Platelet, count | 10^9^/L |
|  | Haemoglobin level (Hb) | g/Dl |
|  | Haematocrit level (HCT) | % |
|  | Mean corpuscular volume (MCV) | fL |
|  | Mean corpuscular haemoglobin (MCH) | Pg |
|  | Mean corpuscular haemoglobin concentration (MCHC) | d/dL |
|  | RBC distribution width (RDW) | % |
|  | Basophil count | 10^9^/L |
|  | Neutrophil count | 10^9^/L |
|  | Eosinophil count | 10^9^/L |
|  | Lymphocyte count | 10^9^/L |
|  | Monocyte count | 10^9^/L |
| Liver function test | Alanine transaminase (ALT) concentration | IU/L |
|  | Alkaline phosphatase (ALP) concentration | U/L |
|  | Albumin concentration | g/L |
|  | Total protein concentration | g/L |
|  | Bilirubin concentration | $\mu$mol/L |
|  | Globulin concentration | g/L |
| Renal function test | Urea concentration | mmol/L |
|  | Sodium concentration | mmol/L |
|  | Potassium concentration | mmol/L |
|  | Creatinine | $\mu$mol/L |
